# Prognostic significance of left atrial strain in sarcomere gene variant carriers without hypertrophic cardiomyopathy

**DOI:** 10.1111/echo.15434

**Published:** 2022-08-17

**Authors:** Roy Huurman, Daniel J. Bowen, Ferit O. Mutluer, Bernardo Loff Barreto, Marjon A. van Slegtenhorst, Judith M. A. Verhagen, Alexander Hirsch, Annemien E. van den Bosch, Michelle Michels, Arend F. L. Schinkel

**Affiliations:** ^1^ Department of Cardiology Thoraxcenter, Erasmus MC University Medical Center Rotterdam Rotterdam The Netherlands; ^2^ Department of Cardiology Yeditepe University Hospital Istanbul Turkey; ^3^ Department of Clinical Genetics Erasmus MC University Medical Center Rotterdam Rotterdam The Netherlands; ^4^ Department of Radiology and Nuclear Medicine, Erasmus MC University Medical Center Rotterdam Rotterdam The Netherlands

**Keywords:** cardiogenetics, echocardiography, hypertrophic cardiomyopathy, left atrial strain, speckle‐tracking echocardiography

## Abstract

**Background:**

Genetic testing of relatives of hypertrophic cardiomyopathy (HCM) patients has led to a large group of genotype‐positive, phenotype‐negative (G+/Ph−) subjects. Prediction of progression to overt HCM in these subjects is challenging. While left atrial (LA) strain is reduced in HCM patients it is currently unknown whether this parameter can be used to predict HCM phenotype progression.

**Methods:**

This study includes 91 G+/Ph− subjects and 115 controls. Standard echocardiographic parameters as well as left ventricular global longitudinal strain (LV GLS) and LA reservoir strain (LASr) were assessed for each patient. Logistic and Cox proportional hazard regression analyses were used to investigate predictors of G+/Ph− status and HCM during follow‐up.

**Results:**

Independent predictors of G+ status included pathological Q waves (OR 1.60 [1.15–2.23], *p* < .01), maximal wall thickness (MWT: OR 1.10 [1.07–1.14], *p* < .001), mitral inflow E wave (OR 1.06 [1.02–1.10, *p* = .001), A wave (OR 1.06 [1.03–1.10], *p* < .001), LV GLS (OR .96 [.94–.98], *p* < .001), and LASr (OR .99 [.97–.99], *p* = .03). In univariable Cox regression analysis, male sex (HR 2.78 [1.06–7.29], *p* = .04), MWT (HR 1.72 [1.14–2.57], *p* = .009) and posterior wall thickness (HR 1.65 [1.17–2.30], *p* = .004) predicted HCM during a median follow‐up of 5.9 [3.2–8.6] years, whereas LASr did not (HR .95 [.89–1.02], *p* = .14). There were no significant predictors of HCM after multivariable adjustment.

**Conclusion:**

LASr is significantly impaired in G+/Ph− subjects and is an independent predictor of G+/Ph− status, but did not predict HCM development during follow‐up.

## 1 INTRODUCTION

Hypertrophic cardiomyopathy (HCM) is the most common hereditary cardiac disease with a prevalence ranging between 1:200 and 1:500. A pathogenic sarcomere gene variant is detected in up to two‐thirds of cases.^1^ Genetic testing of relatives of patients with HCM has led to the identification of a large group of patients who carry pathogenic variants causing HCM, without left ventricular (LV) hypertrophy. Repeated follow‐up of this genotype‐positive, phenotype‐negative (G+/Ph−) group is advised, primarily due to the age‐related penetrance of HCM. A substantial portion of these subjects will remain free of HCM during their lifetime.^2^ Although a myriad of features have been implicated as part of the pre‐clinical phenotype of HCM,^3^, ^4^, ^5^, ^6^ including abnormalities in diastolic function and the presence of myocardial crypts, the natural history of this patient group remains incompletely characterized. As a result, accurately predicting which G+/Ph− subjects will develop overt HCM remains difficult.

Advances in speckle‐tracking echocardiography (STE) have given valuable insights into the mechanical properties of the left atrium (LA) during the cardiac cycle and have demonstrated the clear prognostic value of LA strain abnormalities in several disease states, independent of LA size.^7^, ^8^, ^9^ The LA aids the LV in maintaining cardiac output, and remodels in the setting of LV diastolic dysfunction with elevated filling pressures, as well as in the presence of LV hypertrophy, LV outflow tract obstruction, mitral regurgitation, and possibly by primary atrial alterations as well, ultimately impairing LA function.^10^ This is previously demonstrated in HCM patients using STE.^10^, ^11^ It is possible that LA strain is decreased in the G+/Ph− population as well, as abnormalities in diastolic function are often encountered in this group,^12^, ^13^ We hypothesize that subjects with a more severe pre‐clinical phenotype (i.e., those with reduced LA strain) are more prone to develop HCM later in life. For this study, we measured LA reservoir strain (LASr), which represents the function of the LA as a reservoir for pulmonary venous return during ventricular contraction, reflecting LA compliance as the base of the LV descends during systole. The aims of this study are to quantify and compare LASr in G+/Ph− subjects and in healthy controls and to examine whether it predicts the development of HCM in G+/Ph− subjects.

## 1 METHODS

### 1.1 Study population

For this single‐center retrospective cohort study we screened all subjects with pathogenic sarcomere gene variants. Subjects were eligible for inclusion when at least one transthoracic echocardiographic (TTE) study was performed, demonstrating the absence of HCM. The diagnosis of HCM was based on a maximal wall thickness (MWT) ≥13 mm, in accordance with international guidelines.^1^ Subjects were excluded if other imaging modalities (cardiovascular magnetic resonance [CMR] or computed tomography imaging) performed at the same time as the baseline TTE study revealed discrepant wall thickness measurements reclassifying the subject as an HCM patient or if the location of MWT was not adequately visualized but subjects were highly suspected of a phenotype (i.e., apical HCM). This study conforms to the principles of the Declaration of Helsinki. All patients and controls gave informed consent for inclusion in the registry and local institutional review board approval was obtained.

### 1.2 Control group

The healthy control group consisted of 147 volunteers recruited in 2014–2015 and evaluated at our outpatient clinic, the protocol has been described in detail previously.^14^ Healthy volunteers between 20 and 72 years underwent a routine cardiac assessment consisting of medical history, physical examination, electrocardiography (ECG), and TTE. Subjects were excluded in case of previous or present cardiovascular disease, hypertension, diabetes mellitus, hypercholesterolemia, systemic disease, or use of medication potentially influencing cardiac function or cardiac abnormalities at physical examination or on the ECG.

### 1.3 Clinical assessment

Routine clinical assessment of the G+/Ph− group consisted of medical history, physical examination, ECG, and TTE. All ECGs were evaluated for signs of left ventricular hypertrophy (LVH), defined as a Romhilt‐Estes score ≥4, pathological Q waves (in accordance with the European Society of Cardiology/American College of Cardiology/American Heart Association/World Heart Federation recommendations), and T‐wave inversions.^15^

### 1.4 Echocardiographic analysis

**FIGURE 1:**
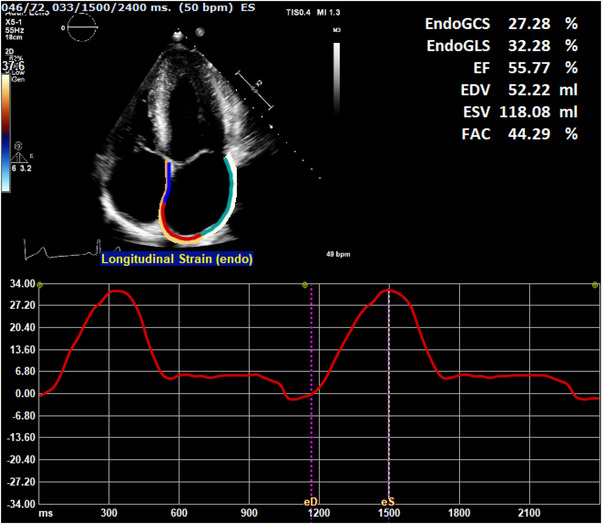
Example of left atrial reservoir strain measurement in a genotype‐positive, phenotype‐negative subject. Top left: Apical four‐chamber view with semi‐automatic overlay of left atrial wall. Bottom: Left atrial strain curve during two cardiac cycles. X‐axis: cardiac cycle in milliseconds, starting from end‐diastole. End‐systole marked during second cycle at 1500 ms. Y‐axis: Strain value (%) relative to the zero strain reference sat at end‐diastole. Top right: data extracted from left atrial wall tracing, *EndoGLS* represents relevant reservoir strain value, corresponding to the difference between peak strain at end‐systole and zero strain at end‐diastole.

TTE studies were performed using an iE33 or EPIQ7 ultrasound system (Phillips Medical Systems, Amsterdam, The Netherlands). All subjects underwent complete TTE studies using a standardized protocol based on current international guidelines.^16^, ^17^ Left ventricular systolic and diastolic function were analyzed according to the guidelines. LV diastolic function was defined as normal, abnormal relaxation, pseudonormal, or restrictive filling, based on Doppler mitral inflow pattern parameters including early (E) and late (A) LV filling velocities, deceleration time, tissue Doppler imaging‐derived septal early diastolic velocities (e’) and left atrial (LA) dimensions. Additional variables used in this study included MWT divided by posterior wall thickness and by body‐surface area indexed LV mass (MWT/PWT and MWT/LVMi).^18^ LV mass was calculated using the Devereux formula.^19^ Echocardiographic images were transferred to a vendor‐independent analysis system (Image Arena version 4.6, TomTec Imaging Systems, Unterschleißheim, Germany), whereby all STE measurements were performed using a chamber‐specific wall motion tracking software (2D‐CPA). Before attempting STE, the feasibility of LA strain analysis was judged by two investigators (RH, DB) by the following criteria: (1) the presence of an adequate apical four‐chamber view with no or minimal foreshortening of the LA; (2) that image quality was sufficient (in regard to orientation, depth, gain and visualization of the endocardial border and mitral annulus), and (3) the cardiac cycle was complete, recorded at least twice and accompanied by an ECG tracing. Once images were deemed adequate, the QRS peak was used as an automated surrogate for end‐diastole,^20^ and the zero reference point was set at this point. End‐systole was marked as the frame prior to mitral valve opening. The left atrial endocardial border was manually traced in the end‐diastolic frame and adjusted if required following automated tracking in the end‐systolic frame. Resultant strain curves were assessed for accuracy when compared to the underlying wall motion, paying special attention to any extrapolated movements across pulmonary veins and the left atrial appendage. LA reservoir phase was defined as the time between ventricular end‐diastole (mitral valve closure) and mitral valve opening. LASr was calculated as the positive strain difference between these two time points (Figure 1). The method employed follows the recommendations outlined in the European Association of Cardiovascular Imaging/American Society of Echocardiography/Industry Task Force consensus document.^21^ Initial LASr measurements were repeated in 20 random subjects approximately 9 months after the initial analysis (DB) and by a second reader (BLB), both with approximately 10 years of experience. LV strain analysis was performed as previously described,^6^ using standard four‐, three‐ and two‐chamber images.

### 1.5 Genetic testing

Genetic counseling and testing is routinely offered to probands with HCM visiting our cardiogenetic outpatient clinic. Subjects are considered G+ in case of a likely pathogenic or pathogenic variant (class 4 or 5) in a HCM‐causing gene, in accordance with the American College of Medical Genetics and Genomics recommendations.^22^ Cascade genetic screening is offered to close relatives targeting the specific variant found in the proband. G+ relatives are then referred for cardiac screening. The cardiogenetic testing procedure has been previously described.^23^

### 1.6 Follow‐up imaging

Time‐to‐event analyses were performed for the development of HCM during follow‐up. Therefore, for each subject, MWT of all follow‐up TTEs was assessed. The endpoint for this analysis was met if the diagnostic criterion for HCM was met (i.e., MWT ≥13 mm) in any subsequent TTE. In subjects that developed HCM, the previous TTE study date (i.e., the last study without HCM) was registered and subjects were interval‐censored between the two study dates, as the actual development of HCM occurred in this interval. Subjects who did not develop HCM during follow‐up were right‐censored on the date of the last TTE. Follow‐up duration was calculated from baseline TTE until date of first TTE demonstrating HCM or until date of last TTE in those without HCM.

### 1.7 Statistical analysis

Values were expressed as mean ± standard deviation, median [25^th^–75^th^ percentile] or number (%). Continuous data were assessed for normality by inspecting Q‐Q plots and using the Shapiro‐Wilk test, and were analyzed using the Student's *t*‐test or Mann–Whitney *U* test, as appropriate. Categorical data were compared using Pearson's χ^2^ test. Univariable and multivariable logistic regression and Cox proportional hazard regression analyses were used to assess the predictive ability of LA strain and other baseline clinical, ECG, and echocardiographic characteristics for G+ status and for the development of HCM during follow‐up, respectively. For the latter, interval‐censoring was done half‐open, half‐closed and 500 bootstrap samples were used. Variable selection was based on comparison of the Akaike Information Criterion. Predictive performance of LASr values is expressed through the calculation of the area under the curve (AUC). Inter‐ and intra‐reader reproducibility was assessed in 20 random subjects using the intraclass correlation coefficient (ICC). All testing was two‐tailed and *p* values < .05 were considered statistically significant. All statistical analyses were performed using SPSS version 25 (IBM Corp., Armonk, New York) and R version 3.6.1 (https://cran.r‐project.org/) using the *icenReg* and *psych* package.

## 2 RESULTS

### 2.1 Clinical characteristics

Of 160 G+/Ph− subjects screened for this study, 91 were ultimately included. One subject was excluded because of concomitant Tetralogy of Fallot, others because of incomplete images (*n* = 8), insufficient image quality (*n* = 49), and inadequate tracking (*n* = 11). An age‐ and sex‐matched population of 115 healthy volunteers were included. Clinical and echocardiographic characteristics for the study population and control group are displayed in Table 1. The most commonly affected gene was MYBPC3 (*n* = 74, 81%). Co‐morbidities and medication use were absent in the control group. Nine study subjects were diagnosed with hypertension (10%). On ECG, LVH criteria (Romhilt‐Estes score ≥4) were met by a small number of subjects in both the study and control group, 7 (8%) versus 4 (4%), respectively (*p* = .22). Pathological Q waves were found only in study subjects, in seven cases (8%).

**TABLE 1:** Clinical characteristics of sarcomere gene variant carriers and control population

Variable	Carriers (*n* = 91)	Controls (*n* = 115)	*p*‐value
Age, years	39 [30–48]	42 [30–55]	.11
Male sex	36 (40%)	49 (43%)	.67
Body surface area, m^2^	1.89 ± .22	1.87 ± .19	.35
Systolic blood pressure, mmHg	120 [110–140]	124 [115–130]	.95
Diastolic blood pressure, mmHg	75 [70–80]	78 [75–84]	.02
**Genotype**			
MYBPC3	74 (81%)	–	–
MYH7	7 (8%)	–	–
Other^a^	10 (11%)	–	–
**Medical history**			
Arterial hypertension	9 (10%)	0 (0%)	.001
Atrial fibrillation	0 (0%)	0 (0%)	–
Diabetes mellitus	1 (1%)	0 (0%)	.44
Hypercholesterolemia	3 (3%)	0 (0%)	.09
**Medical therapy**			
Antihypertensive agents^b^	6 (7%)	0 (0%)	.007
Statins	3 (3%)	0 (0%)	.09
Antithrombotic agents^c^	0 (0%)	0 (0%)	–
Antidiabetic agents	1 (1%)	0 (0%)	.44
**Electrocardiography**			
Sinus rhythm	91 (100%)	118 (100%)	–
Romhilt‐Estes ≥4	7 (8%)	4 (4%)	.22
Pathological Q wave	7 (8%)	0 (0%)	.003
T wave inversion	1 (1%)	0 (0%)	.44

Data are expressed as number (%), mean ± standard deviation or median [25^th^–75^th^ percentile].

^a^Includes MYL2 (*n* = 3), TNNT2 (*n* = 2), and ALPK3, MYL3, MYPN2, TNNI3, TPM1 (*n* = 1 each).

^b^Includes angiotensin‐converting enzyme inhibitors (*n* = 2), angiotensin II receptor blockers (*n* = 2), betablockers (*n* = 2), diuretics (*n* = 1).

^c^Includes antiplatelet and anticoagulant medication.

### 2.2 Echocardiographic results

**FIGURE 2:**
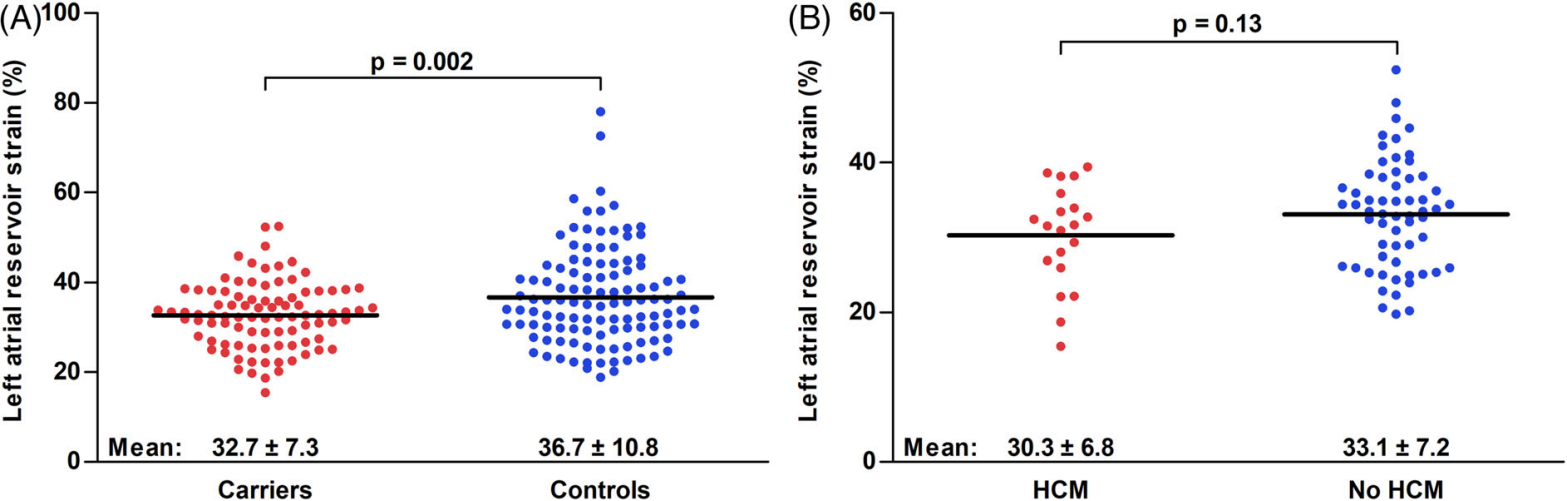
Beeswarm plots of individual left atrial reservoir strain results in genotype‐positive, phenotype‐negative subjects [*carriers*] compared to controls (A), and in genotype‐positive subjects who do or do not develop hypertrophic cardiomyopathy during 5.9 [3.2–8.6] years follow‐up (B). Line indicates mean. HCM: hypertrophic cardiomyopathy.

Table 2 illustrates results from conventional echocardiography and STE. The G+/Ph− population had a 2 mm higher mean MWT, LV mass was higher accordingly (76 ± 17 vs. 62 ± 13 g/m^2^, *p* < .001). Diastolic function was mostly similar, although both the mitral inflow E and A waves as well as the E/e’ ratio were larger in the study group. Both LV GLS as well as LASr were significantly different between the groups (LV GLS: −21.1 ± 2.7% vs. −20.0 ± 2.4%, *p* = .003; LASr: 32.7 ± 7.3% vs. 36.7 ± 10.8%, *p* = .002). Figure 2A illustrates the individual LASr values for both groups. Logistic regression analysis was used to assess whether G+ status could be predicted using clinical characteristics as well as ECG and TTE variables (Table 3). After multivariable adjustment, both LV GLS as well as LASr were predictors of G+ status, together with pathological Q waves and mitral inflow E and A waves. The AUC of LASr for prediction of genotype status was .60 (95% confidence interval .52–.67, *p* = .02). LASr values above 48% achieved a negative predictive value of 89%; LASr values above 53% were found exclusively in genotype‐negative subjects. Conversely, the positive predictive value of LASr values below 22% was 63%.

**TABLE 2:** Echocardiographic characteristics of sarcomere gene variant carriers and healthy controls

Variable	Carriers (*n* = 91)	Controls (*n* = 115)	*p*‐value
**Left ventricular dimensions**			
End‐diastolic diameter, mm	47 ± 5	46 ± 4	.12
Maximal wall thickness, mm	10 ± 2	8 ± 2	<.001
Posterior wall thickness, mm	8 ± 1	8 ± 1	.007
Mass, indexed, g/m^2^	76 ± 17	62 ± 13	<.001
MWT/LVMi, mm/g/m^2^	.13 ± .02	.13 ± .02	.91
MWT/PWT	1.1 ± .2	1.0 ± .2	<.001
**Left ventricular function**			
Impaired systolic function	2 (2%)	0 (0%)	.19
Impaired diastolic function	13 (14%)	16 (14%)	1.00
Mitral inflow E wave, cm/s	78 ± 18	71 ± 16	.004
Mitral inflow A wave, m/s	58 ± 17	48 ± 14	<.001
E/A ratio	1.46 ± .55	1.61 ± .67	.09
Deceleration time, ms	199 ± 49	189 ± 33	.08
Septal e’, cm/s	9.7 ± 2.4	9.7 ± 2.5	.88
E/e’ ratio	8.3 ± 2.0	7.5 ± 1.7	.002
Global longitudinal strain, %	−21.1 ± 2.7	−20.0 ± 2.4	.003
**Left atrium**			
Diameter, mm	36 ± 5	34 ± 4	.001
Reservoir strain, %	32.7 ± 7.3	36.7 ± 10.8	.002

Data are expressed as number (%) or mean ± standard deviation. Abbreviations: LVMi, left ventricular mass indexed for body surface area; MWT, maximal wall thickness; PWT, posterior wall thickness.

**TABLE 3:** Logistic regression analysis for genotype‐positive group status

	Univariable		Multivariable	
Variable	Odds ratio [95% CI]	*p*‐value	Odds ratio [95% CI]	*p*‐value
**Clinical and ECG characteristics**				
Body surface area, per m^2^	2.02 [.46–8.91]	.35		
Romhilt‐Estes ≥4	2.31 [.68–9.07]	.19		
Pathological Q wave	20.50 [2.44–2674.47]	.02	1.60 [1.15–2.23]	.005
T wave inversion	3.83 [.20–561.42]	.37		
**Left ventricular dimensions**				
End‐diastolic diameter, per mm	1.05 [.99–1.12]	.11		
Maximal wall thickness, per mm	1.62 [1.37–1.94]	<.001	1.10 [1.07–1.14]	<.001
Posterior wall thickness, per mm	1.46 [1.17–1.86]	.001		
Mass, indexed, per g/m^2^	1.07 [1.04–1.09]	<.001		
MWT/LVMi, per .01 increase	.99 [.87–1.13]	.91		
MWT/PWT, per .1 increase	1.28 [1.11–1.47]	.001		
**Left ventricular function**				
Impaired systolic function	6.45 [.52–894.33]	.16		
Impaired diastolic function	1.03 [.46–2.27]	.94		
Mitral inflow E wave, per .1 m/s	1.27 [1.08–1.50]	.005	1.06 [1.02–1.10]	.001
Mitral inflow A wave, per .1 m/s	1.52 [1.26–1.86]	<.001	1.06 [1.03–1.10]	<.001
E/A ratio, per unit	.66 [.41–1.05]	.08		
Deceleration time, per ms	1.01 [1.00–1.01]	.08		
Septal e’, per cm/s	.99 [.88–1.11]	.88		
E/e’, per unit	1.27 [1.09–1.50]	.002		
Global longitudinal strain, per %	.84 [.75–.94]	.004	.96 [.94–.98]	<.001
**Left atrium**				
Diameter, per mm	1.11 [1.04–1.19]	.001		
Reservoir strain, per %	.95 [.92–.98]	.004	.99 [.97–.99]	.03

Data are expressed as odds ratio [95% confidence interval]. LVMi was disregarded for multivariable analysis due to incompleteness of the data (*n* = 17).

Abbreviations: LVMi, left ventricular mass indexed for body surface area; MWT, maximal wall thickness; PWT, posterior wall thickness.

### 2.3 Follow‐up

In the G+/Ph− group, 80 (88%) subjects had follow‐up imaging studies. After a median follow‐up of 5.9 [3.2–8.6] years, a total of 20 (25%) G+/Ph− subjects were diagnosed with HCM. Subjects developed HCM after a median follow‐up of 4.1 [2.1–8.2] years, median follow‐up of those that did not develop HCM was 6.2 [3.8–8.7] years. An overview of clinical, ECG and TTE characteristics for subjects with and without HCM during follow‐up is displayed in Table 4. Subjects who were diagnosed with HCM during follow‐up were more often men, had a higher MWT, PWT and LVMi at baseline, but had similar LASr values (Figure 2B). Results of univariable and multivariable Cox proportional hazard regression analysis are shown in Table 5. Male sex (HR 2.78 [1.06–7.29], *p* = .67), MWT (HR 1.72 [1.14–2.57], *p* = .009), and PWT (HR 1.65 [1.17–2.30], *p* = .004) were significant predictors of HCM during follow‐up, whereas LASr was not (HR .95 [.89–1.02], *p* = .14). There were no significant predictors of HCM after multivariable adjustment. The AUC of LASr for the prediction of HCM during follow‐up was .60 (95% CI [.46–.74], *p* = .18).

**TABLE 4:** Baseline and imaging characteristics for genotype‐positive, phenotype‐negative subjects stratified by the presence of HCM during 5.9 [3.2–8.6] years follow‐up

Variable	HCM (*n* = 20)	No HCM (*n* = 60)	*p*‐value
**Clinical characteristics**			
Age, years	44 ± 15	39 ± 13	.12
Male sex	12 (60%)	19 (32%)	.03
Body surface area, m^2^	1.97 ± .28	1.88 ± .20	.23
Systolic blood pressure, mmHg	130 [115–142]	120 [110–135]	.28
Diastolic blood pressure, mmHg	77 [70–85]	75 [70–80]	.67
**Genotype**			.21
MYBPC3	19 (95%)	47 (78%)	
MYH7	0 (0%)	6 (10%)	
Other^a^	1 (5%)	7 (12%)	
**Medical history**			
Arterial hypertension	4 (20%)	5 (8%)	.22
Atrial fibrillation	0 (0%)	0 (0%)	–
Diabetes mellitus	0 (0%)	1 (2%)	1.00
Hypercholesterolemia	0 (0%)	3 (5%)	.57
**Medical therapy**			
Antihypertensive agents^a^	1 (5%)	5 (8%)	1.00
Statins	0 (0%)	3 (5%)	.57
Antithrombotic agentsa	0 (0%)	0 (0%)	–
Antidiabetic agents	0 (0%)	1 (2%)	1.00
**Electrocardiography**			
Romhilt‐Estes ≥4	2 (10%)	1 (2%)	.15
T wave inversion	1 (5%)	0 (0%)	.25
Pathological Q wave	3 (15%)	4 (7%)	.36
**Left ventricular dimensions**			
End‐diastolic diameter, mm	46 ± 6	47 ± 5	.79
Maximal wall thickness, mm	10 [8–11]	8 [7–9]	.001
Posterior wall thickness, mm	8 [7–9]	8 [7–8]	.003
Mass, indexed, g/m^2^	74.5 [65.0–84.1]	61.0 [52.8–70.1]	.04
MWT/LVMi, mm/g/m^2^	.13 [.11–.15]	.13 [.11–14]	.44
MWT/PWT	1.1 [1.0–1.3]	1.0 [.9–1.1]	.57
**Left ventricular function**			
Impaired systolic function	1 (5%)	1 (2%)	.44
Impaired diastolic function	3 (15%)	10 (17%)	1.00
Mitral inflow E wave, cm/s	72 ± 15	78 ± 19	.18
Mitral inflow A wave, cm/s	58 ± 19	59 ± 17	.90
E/A ratio	1.37 ± .54	1.45 ± .55	.60
Deceleration time, ms	206 ± 60	195 ± 47	.35
e’, cm/s	8.8 ± 2.2	9.6 ± 2.4	.17
E/e’ ratio	8.4 ± 1.7	8.4 ± 2.0	.83
Global longitudinal strain, %	−20.8 ± 2.0	−21.1 ± 3.0	.67
**Left atrium**			
Diameter, mm	37 ± 6	36 ± 5	.17
Reservoir strain, %	30.3 ± 6.8	33.0 ± 7.2	.13

Data are expressed as number (%), mean ± standard deviation or median [25^th^–75^th^ percentile].

Abbreviations: LVMi, left ventricular mass indexed for body surface area; MWT, maximal wall thickness; PWT, posterior wall thickness. ^a^See table 1 for relevant genes and drug types.

**TABLE 5:** Cox proportional hazard regression analysis for the development of hypertrophic cardiomyopathy in 80 genotype‐positive, phenotype‐negative subjects during 5.9 [3.2–8.6] years follow‐up

	Univariable		Multivariable	
Variable	Hazard ratio [95% CI]	*p*‐value	Hazard ratio [95% CI]	*p*‐value
**Clinical and ECG characteristics**				
Age, per year	1.03 [.99–1.08]	.14		
Male sex	2.78 [1.06–7.29]	.04	1.38 [.30–6.29]	.67
Body surface area, per m^2^	5.47 [.20–146.22]	.31		
Systolic blood pressure, per mmHg	1.02 [.99–1.06]	.15		
Diastolic blood pressure, per mmHg	1.01 [.95–1.07]	.79		
Arterial hypertension	2.80 [.10–77.51]	.54		
Pathological Q wave	2.94 [.01–1191.93]	.72		
**Left ventricular dimensions**				
End‐diastolic diameter, per mm	.96 [.85–1.08]	.48		
Maximal wall thickness, per mm	1.72 [1.14–2.57]	.009	1.54 [.87–2.74]	.14
Posterior wall thickness, per mm	1.65 [1.17–2.30]	.004	1.14 [.63–2.05]	.66
Mass, indexed, per g/m^2^	1.02 [1.00–1.05]	.10		
MWT/LVMi, per .01 increase	1.16 [.92–1.46]	.20		
MWT/PWT, per .1 increase	1.22 [.92–1.63]	.16		
**Left ventricular function**				
Impaired diastolic function	1.27 [.01–112.10]	.92		
Mitral inflow E wave, per .1 m/s	.88 [.71–1.09]	.24		
Mitral inflow A wave, per .1 m/s	.99 [.69–1.43]	.97		
E/A ratio, per unit	.87 [.30–2.53]	.80		
Deceleration time, per ms	1.01 [1.00–1.02]	.23		
Septal e’, per cm/s	.88 [.69–1.13]	.33		
E/e’, per unit	1.00 [.80–1.26]	.97		
Global longitudinal strain, per %	1.07 [.93–1.23]	.37		
**Left atrium**				
Diameter, per mm	1.06 [.96–1.19]	.28		
Reservoir strain, per %	.95 [.89–1.02]	.14		

Data are expressed as hazard ratio [95% confidence interval]. Due to data separation, genotype, medical history (except arterial hypertension), medical therapy, Romhilt‐Estes score, T wave inversion and systolic function were not entered as independent variables.

Abbreviations: LVMi, left ventricular mass indexed for body surface area; MWT, maximal wall thickness; PWT, posterior wall thickness.

### 2.4 Inter‐ and intra‐reader reproducibility

**FIGURE 3:**
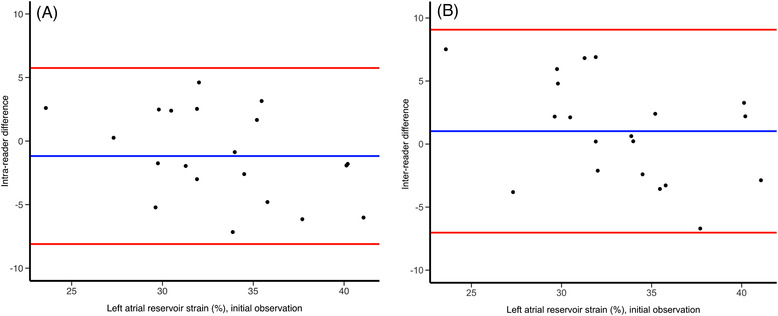
Bland‐Altman plots demonstrating intra‐ and inter‐reader reproducibility. The x axis demonstrates initial left atrial reservoir strain values, plotted against intra‐ (A) and inter‐reader differences (B) for 20 random subjects. Blue line: mean difference; red lines: limits of agreement (mean ± 2 standard deviations).

Mean intra‐ and inter‐reader differences for measurements repeated in 20 random subjects were −1.2 ± 3.5% and 1.0 ± 4.1%, respectively. Intra‐reader and inter‐reader reproducibility were .68 [.41–.84] and .58 [.28–.78], indicating moderate agreement. The corresponding Bland‐Altman plots are shown in Figure 3.

## 3 DISCUSSION

This study demonstrated that LASr is decreased in G+/Ph− subjects compared to controls, demonstrating functional alterations before the onset of an overt HCM phenotype. However, LASr is similar in G+/Ph− subjects who develop HCM compared to those who do not, and does not predict HCM during follow‐up.

To our knowledge, this is the first study investigating LA strain in G+/Ph− subjects. Many studies have reported on supposed pre‐clinical markers of HCM, which include diastolic dysfunction, myocardial crypts, mitral valve leaflet abnormalities and changes in T1 and extracellular volume mapping on CMR.^3^, ^4^, ^5^, ^12^, ^18^ The current study demonstrates statistically significant differences in LASr values in G+/Ph− subjects compared to healthy controls. The overlap in LASr values between the groups is profound in both comparisons, which fits with the low predictive performance of LASr and the lack of an association in Cox proportional hazard regression. Higher LASr values (i.e., above 53%) were found only in controls, indicating that there is some potential in using LASr to identify those least likely to be sarcomere gene variant carriers. These data suggest that carriers undergo subtle functional changes before the onset of a clear HCM phenotype as well as before changes measurable by conventional echocardiography, which is evident from their largely preserved systolic and diastolic LV function. Regretfully, the large overlap between groups hinders the clinical application of these differences. Similarly, LASr cannot be used to further stratify G+/Ph− subjects at risk of developing HCM later in life. A similar pattern was seen with LV GLS, which was increased in G+/Ph− subjects but also did not predict HCM, which is in line with earlier research conducted in our center.^6^ The association of MWT with genotype‐status and HCM during follow‐up reflects clinical practice, in which subjects with borderline LVH will be monitored more closely. Our study demonstrates, similar to others, the association of pathological Q waves with genotype‐status.^3^, ^6^ In contrast to van Velzen et al., our study found no association with Q waves and HCM during follow‐up. The discrepant results likely stem from small samples sizes in both studies, as is clear from the large confidence intervals, which stresses the need for larger‐scale studies investigating these and other features in the prediction of HCM. Diastolic indices (mitral inflow E and A and E/e’) predicted genotype‐status as well, although the clinical value is similarly low. Particularly for E/e’, virtually all measurements were within normal limits. Observations of diastolic abnormalities in the pre‐clinical stage of HCM have been made previously,^3^, ^6^, ^12^ but so far have not led to methods to predict genotype‐status or HCM during follow‐up. This study shows that left atrial functional changes occur before the presence of overt LVH, and that it thus belongs to the spectrum of preclinical alterations found in the G+/Ph− population. Regretfully, the otherwise negative findings of our study show that at present, there is no place for LASr measurements in clinical care for this particular group.

Reduced LASr is expected in pre‐clinical HCM for a number of reasons. Most histopathological data on HCM focuses on ventricular myocardial tissue samples, but there is also evidence for atrial involvement in patients with HCM. The occurrence of myocyte disarray and hypertrophy as well as interstitial fibrosis in the left and right atrial walls of a subject recently illustrated by Keane et al. implicates a primary atrial myopathy as part of the HCM phenotype.^24^ Clinical evidence suggesting primary atrial alterations in HCM includes observations that a subset of HCM patients exists which only exhibit atrial fibrillation as part of their disease, in the absence of heart failure symptoms and without dilated left atria.^25^, ^26^ The presence of atrial fibrosis is suggested by a high rate of recurrence of atrial fibrillation following pulmonary vein isolation in HCM patients.^27^ Furthermore, a reduction in LASr reflects the LA's response to increased LA afterload secondary to poor LV compliance. Given that the latter is observed in sarcomere gene variant carriers without hypertrophy,^6^, ^12^ this offers an explanation for the current study's observations as well.

This study has several limitations. The study population was relatively small, particularly when comparing G+/Ph subjects with and without HCM. The vast majority of subjects had variants in the MYBPC3 gene, and study results might not be applicable to cohorts comprising different sarcomere gene variants. Many TTE studies were performed in an era in which the LA was not particularly focused on. However, we excluded studies with suboptimal visualization of the LA. Importantly, even though measurements were performed by experienced sonographers, intra‐ and inter‐reader agreement of LASr measurements was only moderate. We cannot exclude the possibility of non‐dedicated LA images interfering with the reliability of measurements even though we excluded many, and also speculate that software dedicated to LA strain analysis will improve this. While studies with suboptimal quality for strain measurements were excluded, comparison of LASr and LV GLS values with those from other studies must be done with caution, given the differences in vendors and methodologies.

## 4 CONCLUSION

LASr is decreased in G+/Ph− subjects compared to healthy controls and independently predicts G+/Ph− status. LASr values were similar in G+/Ph− subjects who do and do not develop HCM during follow‐up, and cannot be used to predict the occurrence of HCM.
